# Glass Ionomer Subgingival Matrix Technique to Restore a Tooth with Severe Root Resorption for Implant Site Development

**DOI:** 10.1155/2020/6676764

**Published:** 2020-11-21

**Authors:** Ahmad Y. Imam, Raghad A. Al-Dabbagh

**Affiliations:** ^1^Oral and Maxillofacial Prosthodontics Department, Faculty of Dentistry, King Abdulaziz University, Jeddah, Saudi Arabia; ^2^Oral and Maxillofacial Rehabilitation Department, Faculty of Dentistry, King Abdulaziz University, Jeddah, Saudi Arabia

## Abstract

Here, we present the multidisciplinary, patient-specific management of a patient with severe external root resorption and bone loss in a maxillary anterior tooth. The tooth was provisionally noninvasively restored with glass ionomer subgingival matrix in preparation for forced orthodontic extrusion, papillary preservation, and implant placement. This approach enables clinicians to control infection within and around the resorbed tooth and then to use it as an anchor for slow forced tooth eruption to correct bone and mucogingival deformities. Aesthetic and functional outcomes were clinically and radiographically satisfactory. The advantages and disadvantages of this technique are discussed.

## 1. Introduction

Trauma to the face and dentition is common and can often have significant repercussions for the individual [1]. The consequences of such trauma can include immediate or delayed aesthetic problems, with or without underlying infections [2]. To achieve predictable outcomes, the management should be timely, patient-specific, and multidisciplinary [3, 4]. Additionally, such patients will need life-long regular follow-up to prevent or manage complications for optimal aesthetics and function [4].

One complication of traumatic dental injury is external root resorption. Injury to the tooth may lead to displacement or removal of the protective precementum layer followed by colonization with multinucleated cells along the root surface. These cells then initiate the resorptive process and may continue to do so depending on the presence of inflammation or pressure [5]. Early diagnosis is sometimes difficult, because this type of resorption progresses slowly and can initially be asymptomatic. However, clinical presentations such as pink tooth discoloration, localised irregularities in gingival contour, and oedematous gingival tissue, with or without gingival bleeding on probing, could be indicative of external root resorption [6]. Management of such lesions depends on the etiological factor or the source of continuous infection. For example, if the source of infection was the pulp, then the recommendation is intracanal medication with calcium hydroxide for 6-12 months [7]. However, delayed or incorrect treatment may lead to severe root resorption that would deem the tooth nonrestorable.

Another complication of dental trauma and associated infection is loss of the supporting tooth structure (bone and soft tissue), which may compromise aesthetic and functional outcomes of treatment. Augmenting horizontal bony defects may be more predictable than the less predictable management of vertical bone loss [8]. Nonsurgical enhancement of bone and soft tissue with forced slow orthodontic extrusion of hopeless teeth may predictably improve bone and soft tissue volume, thus enhancing final restoration aesthetics [9]. Furthermore, this increased vertical bone height and width may further improve the positioning and stability of immediately placed implants and the subsequent outcome [10].

Here, we describe a case of reestablished aesthetics and function in a patient with long-standing severe root resorption due to trauma to a maxillary central incisor. The tooth was provisionally nonsurgically restored with a glass ionomer (GI) subgingival matrix (which we term the “Imam matrix”) in preparation for forced orthodontic extrusion, papillary preservation, and implant placement.

## 2. Case Description

**Figure 1:**
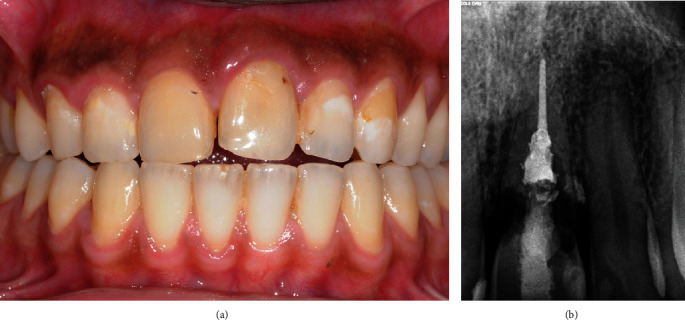
Preoperative clinical and radiographic presentation of tooth #21. (a) Intraoral frontal view shows associated gingival recession and an uneven incisal plane. (b) Periapical radiograph shows extensive root resorption affecting the coronal third of the root.

A 45-year-old man was referred by a periodontist colleague at a private practice (UniDents, Jeddah, Saudi Arabia). The patient reported severe mobility, bleeding, and pain related to the maxillary left front tooth and a history of trauma ten years ago. There had been several attempts at root canal treatment by general dentists and endodontists. Clinically, tooth #21 had grade III mobility, recession, a 7 mm pocket, and soft tissue inflammation. He had a high smile line, uneven anterior incisal plane, and a class I incisal relationship (Figure 1(a)). Radiographically, tooth #21 was root canal treated, had communicating root resorption affecting the coronal third of the root, and interproximal, labial, and palatal bone resorption (Figure 1(b)).

After obtaining patient consent, a multidisciplinary treatment plan was commenced. The plan for tooth #21 included (1) nonsurgical removal of all pathologic tooth structure; (2) nonsurgical provisional restoration of the tooth by creating a subgingival glass ionomer (GI) matrix (“Imam matrix”), cemented fiber post, and composite core; (3) fabrication of a provisional crown and definitive cementation; (4) forced orthodontic tooth extrusion; (5) extraction of the tooth; (6) immediate implant placement; and (7) prosthetic restoration.

**Figure 2:**
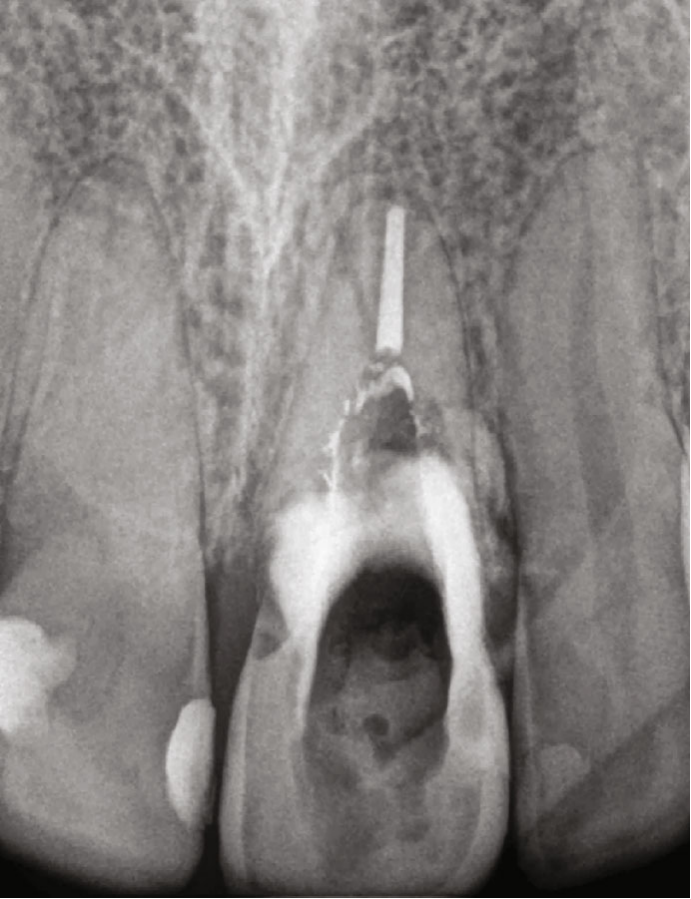
GI subgingival “Imam matrix” that sealed the resorptive root defect.

A putty index (Virtual, Ivoclar Vivadent) was taken prior to tooth preparation, and local anesthesia (lidocaine 2%, Lignospan® standard) was administered. Next, all pathological tooth structures were removed with a diamond bur (gold bur, KOMET) including the existing defective restoration. Gutta percha was removed with a fiber post drill (medium size, 3M, Saint Paul, MN) to leave 3-4 mm at the apical area of the root canal. Bleeding was minimized with a saline-soaked cotton pellet placed on the palatal aspect of the tooth and left for a couple of minutes. Then, paper points were placed into the canal to dry the canal initially and then to maintain canal patency. Next, GI restoration (Fuji IX, GC America Inc., Alsip, IL) was placed palatally, packed with an alcohol swab, and shaped with a plastic filling instrument (HF-PFIDD56, Henry Schein Australia) to build the missing proximal and palatal walls of the root and crown. To ensure bonding of the GI restoration to the deep cervical root margins, it was properly packed with an alcohol swab. Once the GI had set, the paper points were removed from the canal and an access cavity was prepared to leave a 1 mm subgingival band of GI isolating the canal space from the surrounding tissue (fiber post drill; 3M) (Figure 2).

The canal was then acid etched with phosphoric acid following the manufacturer's instructions (Scotchbond™ Universal Etchant, 3M ESPE), rinsed, and dried in preparation for fiber post placement. Dual-cure, self-adhesive resin cement was injected into the canal (RelyX™ Unicem, 3M), and the fiber post (3M) was placed in position. Excess was removed, and the cement was light cured following the manufacturer's instructions. The tooth was then prepared (S6882, KOMET), ensuring all margins were placed at sound tooth structure. Then, the putty index was used to prepare the provisional crown (Protemp, 3M). Video 1 shows step-by-step the process of fabricating the GI matrix. The provisional crown was finished, polished (Enhance Finishing discs, Dentsply Sirona), and cemented with polycarboxylate cement (Poly-F Plus cement BONDEXE, Dentsply). Excess cement was removed, and occlusion was checked.

**Figure 3:**
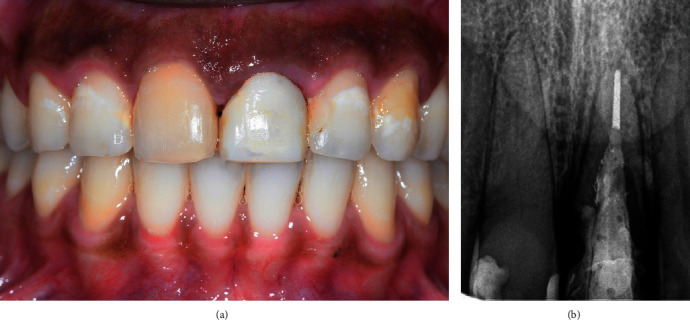
Clinical and radiographic presentation after orthodontic extrusion of tooth 21. (a) Intraoral frontal view showing the coronal relocation of the gingival margin and preservation of interdental papillae. (b) Periapical radiograph showing the coronal repositioning of the apex of the tooth and bone fill proximally.

The patient was then referred to an orthodontist to perform slow orthodontic extrusion. Extrusion of tooth #21 took seven months, including two months for retention. Clinical and radiographic images suggested sufficient formation of bone and soft tissue (Figures 3(a) and 3(b)). The labial gingival recession at tooth 21 was reduced to a level that was satisfactory to the patient.

**Figure 4:**
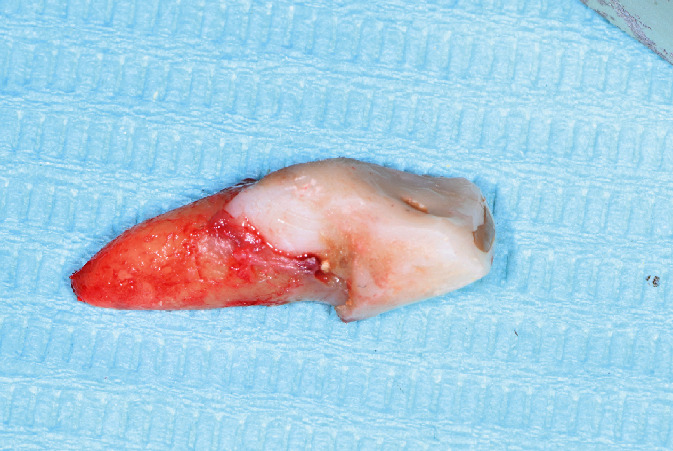
Extracted tooth 21 with well-adapted deep margins of the provisional restoration.

**Figure 5:**
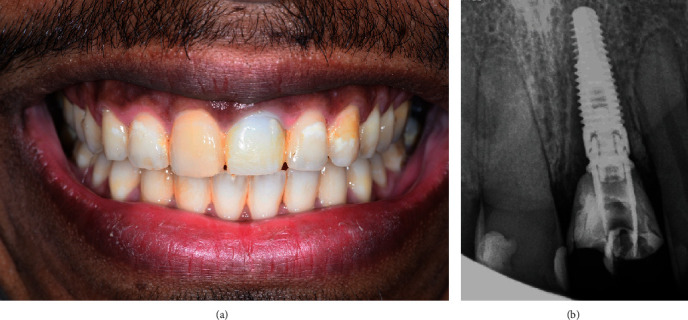
Clinical and radiographic presentation of the immediately placed implant with provisional restoration for tooth #21. (a) Intraoral frontal view. (b) Periapical radiograph.

**Figure 6:**
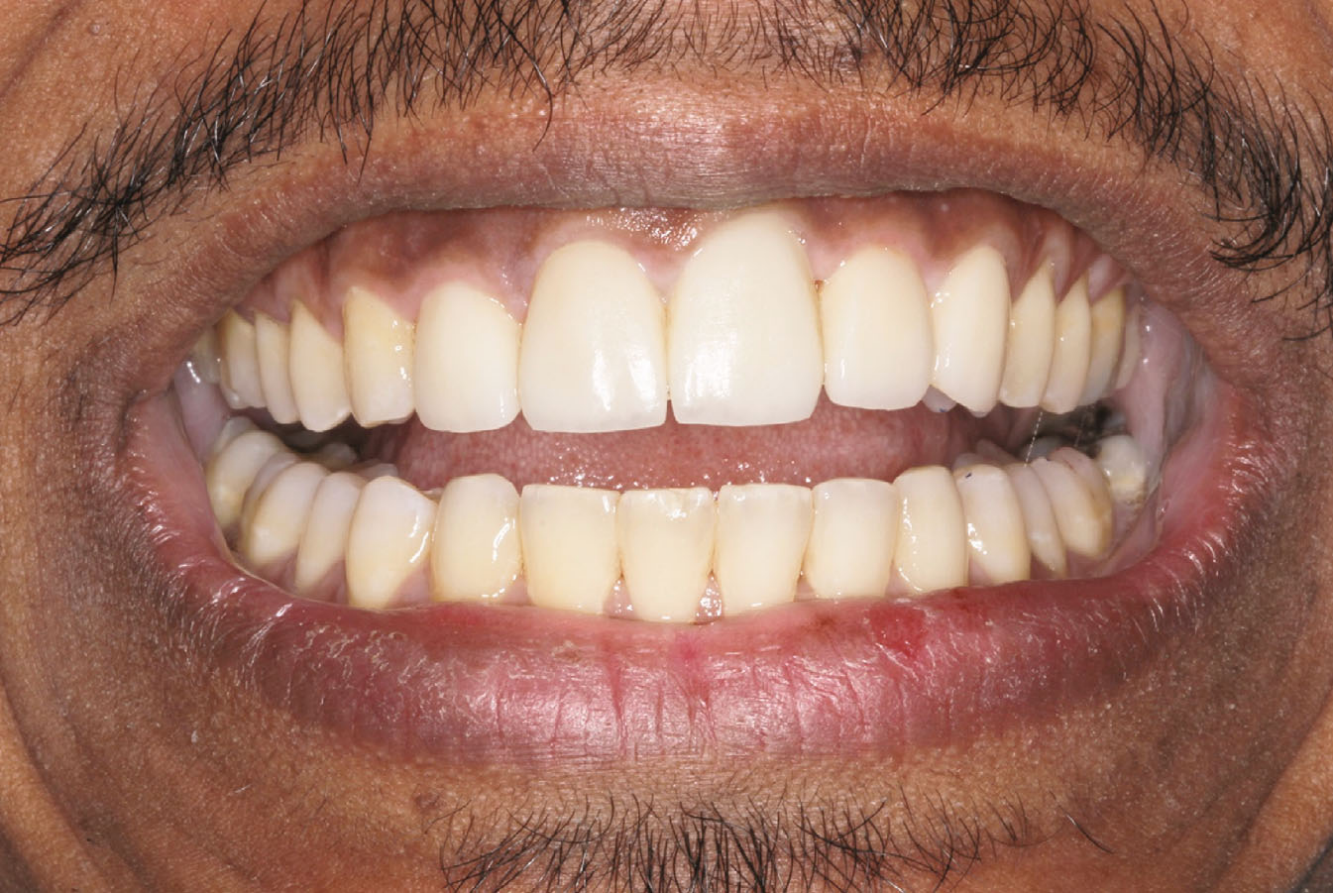
Postoperative clinical presentation of the definitive implant-supported crown of tooth 21.

After achieving satisfactory nonsurgical bone and soft tissue augmentation around tooth #21, the patient was referred back to his periodontist for atraumatic extraction of the tooth (Figure 4) and immediate implant placement (Keystone, Lifecore) and loading with a provisional crown (Figures 5(a) and 5(b)). Six months later, a definitive screw-retained zirconia crown was fabricated (dynamic abutment solution) (Figure 6). The patient maintenance protocol included biannual appointments for clinical and radiographic assessment.

## 3. Discussion

Here, we restored the aesthetics and function of a traumatized tooth by provisionally repairing a nonrestorable maxillary central incisor with severe root resorption for implant site development. In this patient, the central maxillary incisor had recession, mobility, and severe communicating root resorption affecting the coronal third of the root. The treatment plan involved provisionally restoring the tooth, correcting supporting hard and soft tissue volume through slow forced orthodontic extrusion, extraction of the tooth, and finally implant placement. This approach was chosen for interdental papillae preservation, predictable vertical bone augmentation, and achieving a fixed, aesthetically and functionally pleasing restoration throughout treatment.

Restoring a central maxillary incisor with a single implant is aesthetically challenging [11], even more so when there is significant horizontal and vertical bone loss. The main objective is to achieve an osseointegrated dental implant surrounded by healthy soft and hard tissue and placed in a position that enables the dentist to mimic contralateral tooth gingiva position and contour, interdental papillae, and crown shape and shade [11]. Preprosthetic improvement of implant site topography can be achieved either surgically or nonsurgically. Surgical augmentation could include guided tissue regeneration [12], bone and soft tissue graft procedures [13], ridge splitting [14], and distraction osteogenesis [15]. However, surgical augmentation procedures are invasive and painful, can be associated with bleeding and infections, and produce uncertain outcomes when restoring vertical bony defects [13].

In contrast, nonsurgical augmentation involves slow forced orthodontic eruption of nonrestorable teeth, which is predictable and noninvasive, needs patient compliance, and is time consuming [9, 16]; in this case, the patient's exacting personality helped ensure compliance. During orthodontic extrusion, bone and gingival volume increase as a result of stretching of periodontal fibers. These fibers elongate, and osteoblastic cells lay down bone. When this force is slow, gingival tissue follows the newly deposited bone [17].

To use nonrestorable teeth to prepare implant sites, the aim is to eliminate infection prior to noninvasive orthodontic bone and soft tissue augmentation. This patient's central maxillary incisor had severe communicating root resorption affecting the entire coronal third of the root, and there was a proximal and palatal bone loss. Access to the infected part of the root was achieved nonsurgically to remove all infected tooth structure. Then, the resorbed part of the root was sealed with a GI restoration to create the Imam matrix. GI restorations are commonly used to repair external root resorption [18, 19]. The tooth was then reenforced with a fiber post, composite core, and a provisional crown that was definitively cemented. Accessibility and hemorrhage control were challenging but manageable with the aim of controlling infection and enabling the use of this nonrestorable tooth as an anchor for forced extrusion.

The central incisor was previously root canal treated, and the apical root canal treatment was satisfactory with no associated apical radiolucency. Accordingly, after sealing the root defect with the GI restoration, the canal was accessed coronally and the remaining gutta percha was removed to retain the apical 3-5 mm intact. The canal was shaped and irrigated before placement of the fiber post and composite core. Seven months later (orthodontic extrusion), all the surrounding bone tissue looked radiographically normal with no signs or symptoms of infection. Thus, retaining the existing apical seal in this case was sufficient to prevent bacterial percolation into the apical area.

One main advantage of this approach is that it achieves a fixed, aesthetically and functionally pleasing restoration throughout treatment. In this patient, the central incisor suffered from severe mobility and bleeding due to the extensive root resorption that led to the separation of the crown from the root. The provisional restoration of such a tooth was achievable after fabricating the Imam matrix and strengthening it with a fiber post and provisional crown. The tooth was retained until the site was ready for immediate implant placement with an interim crown.

However, this technique might be challenging to achieve. Visual accessibility and hemorrhage control might hinder the bonding of the GI restoration to deep sound tooth margin. If a proper seal and bonding had not been achieved, then extraction with surgical socket preservation methods and bone and soft tissue augmentation techniques would have been alternative options. Indeed, the surgical approach has been used with a predictable outcome when bone surrounding the tooth was preserved post extraction [20]. However, in this patient, there was a significant vertical bone loss from the onset, reaching the middle third of the root, and using surgical bone and soft tissue augmentation techniques would have a compromised aesthetic outcome [13].

Although this approach was time consuming, expensive, multidisciplinary, and highly dependent on patient compliance, it produced a predictable and satisfying aesthetic and functional long-term outcome. Yet, the surgical approach would as well be time consuming and expensive when staged. To our best knowledge, this is the first report to show that provisionally and nonsurgically restoring teeth with extensive root resorption can be achieved by fabricating a GI subgingival “Imam matrix,” fiber post, composite core, and provisional crown. This provisional restorative technique created an infection-free environment and a sufficiently strong tooth to withstand forced eruption for implant site development.

## 4. Summary

This case illustrates the importance of a timely, patient-specific, and multidisciplinary treatment plan. Our management allowed the patient to transition from tooth to implant-supported crown without the use of a removable partial denture. Additionally, a satisfactory aesthetic and functional outcome, with minimal surgical intervention, was achieved.

## Data Availability

Data will be made available on request.

## Abbreviations

GI: Glass ionomer.

## Supplementary Materials

Supplementary Materials Video 1: step-by-step fabrication of the Imam matrix. Image: still image for the video that shows step-by-step the fabrication of the Imam matrix.
