# Supplementary figures and images for: Glass Ionomer Subgingival Matrix Technique to Restore a Tooth with Severe Root Resorption for Implant Site Development
Source: Case Rep Dent. 2020 Nov 21;2020:6676764. doi: 10.1155/2020/6676764 (PMC7704198; doi:10.1155/2020/6676764)

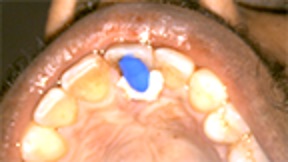

Supplement: Supplementary Materials — Video 1: step-by-step fabrication of the Imam matrix. Image: still image for the video that shows step-by-step the fabrication of the Imam matrix. [file 6676764.f1.zip › 6676764.f1/Still image of video.jpg]
